# Re-focusing the ethical discourse on personalized medicine: a qualitative interview study with stakeholders in the German healthcare system

**DOI:** 10.1186/1472-6939-14-20

**Published:** 2013-05-24

**Authors:** Sebastian Schleidgen, Georg Marckmann

**Affiliations:** 1 Institute of Ethics, History and Theory of Medicine, Ludwig-Maximilians-University Munich, Lessingstrasse 2, Munich, 80336, Germany

## Abstract

**Background:**

In recent years, personalized medicine (PM) has become a highly regarded line of development in medicine. Yet, it is still a relatively new field. As a consequence, the discussion of its future developments, in particular of its ethical implications, in most cases can only be anticipative. Such anticipative discussions, however, pose several challenges. Nevertheless, they play a crucial role for shaping PM’s further developments. Therefore, it is vital to understand how the ethical discourse on PM is conducted, i.e. on what – empirical and normative – assumptions ethical arguments are based regarding PM’s current and future developments.

**Methods:**

To gather this information, we conducted a qualitative interview study with stakeholders in the German health care system. Our purposive sample included 17 representatives of basic research, clinical research, health economics, regulatory authorities, reimbursement institutions, pharmaceutical industry, patient organizations, as well as clinicians and legal experts involved in PM developments or policy making. We used an interview guide with open-ended questions and analyzed transcriptions of the interviews by means of qualitative content analysis.

**Results:**

The respondents addressed a multitude of concerns in the context of research on as well as application of personalized preventive and therapeutic measures both on the individual and on the societal level. Interestingly, regarding future developments of PM the ethical evaluation seemed to follow the rule: the less likely its application, the more problematic a PM measure is assessed. The more likely its application, on the other hand, the less problematic it is evaluated.

**Conclusions:**

The results of our study suggest re-focusing the ethical discourse on PM in Germany towards a *constructive* ethical monitoring which ensures to include *only*, nevertheless *all* of the actual and/or potential concerns that are *ethically relevant* in order to allow balancing them against the actual and potential ethically relevant benefits of PM measures. To render this possible, we propose a strategy for evaluating ethical concerns in the context of PM.

## Background

In recent years, so-called personalized medicine (PM) has become a highly regarded line of development in modern medicine. Basically, this trend results from the overarching goal of PM to develop tailored approaches of prevention and treatment for certain patient subpopulations. The basic concept thereby consists of identifying genetic, phenotypic, or environmental factors, which affect the subpopulation’s health risks and help to find the most appropriate type and dose of medication and/or intervention. In detail, PM promises the following benefits: [1-4] first, it is expected to permit an early identification of persons who carry a genetic or phenotypic variation that increases their risk for developing a certain disease. This would allow preventive measures before disease outbreak. Second, possibilities of better diagnostic and prognostic assessment of diseases are expected resulting in earlier and improved therapeutic interventions. Third, PM is anticipated to develop more effective and safer treatment strategies based on increased knowledge about individual determinants of diseases. Finally, an identification of patient subgroups, which will benefit from a certain therapy, is awaited. This could result in reduced trial and error prescribing with improved effectiveness and efficiency of care.

However, approaches of PM raise several ethical concerns: [1-3,5-10] first, with regard to *research* on PM, measures of adequately implementing informed consent for biomarker studies is discussed as well as issues of confidentiality, data protection and the individuals’ right to know/not to know. Furthermore, it is debated how to allocate resources into as well as within different fields of research on PM in ethically justified ways. Second, when looking at the *clinical usage* of PM measures, concerns are expressed that *predictive test* results may influence individual well-being negatively, increase individual responsibility for one’s health or lead to (genetic) discrimination of persons with predispositions for certain diseases. As regards personalized *diagnostic test* results, worries are articulated that individuals with a certain response rate to a drug could be (genetically) discriminated. Moreover, in reference to personalized *therapeutic measures*, it is suspected that establishing small patient subgroups may lead to insufficient drug testing before their application and thus result in higher risks for patients. Both with regard to personalized prevention and personalized treatment, issues of confidentiality and data protection as well as the individual’s right to know/not to know are discussed. Finally, it is argued that PM measures may lead to significant cost increases and thus result in an additional financial burden for the health care systems. This, it is suspected, could further exacerbate problems of equal access to health care services.

However, PM, understood as biomarker-based targeted treatment or prevention, is still a relatively new field: in Germany, for instance, only 27 drugs are currently authorized for personalized treatment, i.e. their application is based on preceding diagnostic tests on efficacy or (potential) side effects [11]. Meaningful numbers for predictive tests or preventive measures do not exist. Therefore, analysis of PM’s future development, in particular of its opportunities and risks as well as its ethical implications, in most cases can only be anticipatory. Such anticipative discussions, however, pose several challenges, primarily the risks of debating irrelevant ethical concerns or – more importantly – neglecting relevant ones, if debates are not based on a realistic assessment of current and future developments. Nevertheless, such debates play a crucial role for shaping PM’s further developments. Therefore, it is vital to understand how the ethical discourse on PM is conducted, i.e. on what – empirical and normative – assumptions ethical arguments are based regarding PM’s current and future developments. Additionally, it is highly relevant for an adequate ethical monitoring of PM which ethical implications are considered relevant – and which are neglected. Researchers and clinicians who are involved in the development of PM can provide valuable insights both into the current state of development of PM and into the ethical discourse on PM. Furthermore, an examination of ethical concerns requires input from stakeholders of the health care system in which a PM measure is developed and/or used. To gather this important information, we conducted a qualitative interview study with stakeholders in the German health care system.

In detail, the study aimed at answering the following questions: What is the current state of developments of PM? What prospects does PM offer according to the stakeholders’ opinions, i.e. what are the medical areas in which they expect the most significant progress? Which areas are considered less important? What ethical implications of PM are expected, in general and more specifically for the German health care system? The results of this study provide a clearer picture of the perspectives of the rapidly growing field of PM. Moreover, they suggest re-focusing the ethical discourse on PM in Germany, especially regarding a realistic assessment of PM’s current state of development and clinical usage as well as expected future developments.

## Methods

### Sample

To collect as many different stakeholder perspectives as possible, we selected participants in accordance to the criterion of maximum variation regarding their professional role in the German health care system. The purposive sample [12] included representatives of basic research, clinical research, health economics (including statisticians), regulatory authorities, reimbursement institutions, pharmaceutical industry, patient organizations, as well as clinicians and legal experts involved in PM developments or policy making. Leading representatives of each stakeholder group were selected and contacted by mail. The letters included an outline of the project objectives as well as a request for cooperation and informed consent. Ethics approval was not necessary as our study only included experts in the field of PM and did neither endanger the respondents’ psychological or physical health (MBO-Ä §15.1) nor contain any drug or medical device research (AMG §40.1, MPG §20.1). Participants were recruited until a point of saturation was reached, i.e. when the additional interviews provided no further information. Saturation was reached after interviewing 17 participants (Table 1).

**Table 1:** **Participants**: **professional roles**

**Occupation**	**Number**	**Ratio**
Basic Research (BR)	2	12%
Clinical Research (CR)	1	6%
Health Economics (HE)	3	18%
Regulatory Authorities (RA)	1	6%
Reimbursement Institutions (RI)	1	6%
Pharmaceutical Industry (PI)	4	24%
Patient Organizations (PO)	1	6%
Clinicians (C)	2	12%
Legal Experts (LE)	2	12%
**Sum**	**17**	**100**%*

* Difference in Sum due to Rounding.

### Data collection and analysis

We conducted semi-structured interviews based on an interview guide with open-ended questions on the stakeholders’ assessments of current trends and developments as well as their expectations and concerns regarding PM. Except for two interviews, which took place at the interviewers’ office, all conversations were carried out at the respective participants’ office. The interviews took about one hour each. All conversations were held between May and July 2011 and conducted in German, consistently by the same interviewer (SS; trained in qualitative methods). They were audio recorded; the interview guide was adapted to the current state of findings at several stages of the study.

In order to render this adaptation possible as well as to determine the point of saturation, data collection and data analysis were carried out simultaneously. The interviews were transcribed and analyzed following the qualitative content analysis according to Mayring [13] with the software MAXqda. For this purpose, the interview passages relevant to our research questions were identified and coded in order to develop generalizations of the individual statements and conclude overarching categories (Additional file 1). Codes and categories were validated internally (by intercoder consensus) as well as externally (by discussion with experts in the field of qualitative research). As all interviews were conducted in German, the interview passages quoted below are direct translations of the originals. To ensure the least possible bias the passages were translated by an English native speaker.

## Results

The results are divided into three sections: first, the stakeholders’ assessments of the current state of research as well as the clinical use of PM measures are presented. The second section depicts the respondents’ expectations regarding future developments of PM. Finally, their ethical concerns are presented.

### Current state of PM

#### Personalized therapeutic interventions

Overall, the stakeholders were rather skeptical with regard to the current state of research and clinical use of personalized therapeutic measures. One respondent even questioned the scientific basis of PM and considered the underlying idea as inadequately reductionist, i.e. slanted toward molecular factors:

(1) (RI) Momentarily, there is actually nothing that speaks in favor of this being more than just humoralism or something like that. It goes a little further, in that it differs in its biotechnological foundations […], but I initially doubt the whole thing […] for the same reason that basically all other assumptions, which have constituted the basis of a theoretical body with this absoluteness in medicine, turned out to be false.

According to this respondent, the current emphasis on PM is primarily driven by the economic interests of the pharmaceutical industry:

(2) (RI) This is primarily a propagandistic idea by the pharmaceutical industry, which recognized that one could achieve increasingly higher prices for niche products.

Other participants were less critical, but still rather skeptical: Although several personalized therapeutic measures are already applied, their added therapeutic benefit was considered rather small compared to standard interventions:

(3) (C) The [classical chemotherapy] is not that much worse in many cases. There is not a large discrepancy and many patients often are too optimistic on what antibodies and modern cancer therapies can achieve. The two only differ by a few months [of survival].

One respondent additionally considered insufficient possibilities of data management as a major problem. Surprisingly, another respondent suggested that economic interests could be a major reason why PM is not further advanced in its development:

(4) (PO) We also noticed that, conspicuously, the pharmaceutical industry, and this is just a feeling, I do not have any evidence that this is true, is slowing the whole process down. […] One could assume that the pharmaceutical industry thinks along the lines of: well, if we support research efforts then we are risking that the sales volume for substance X will not remain as large because it will be implemented selectively.

#### Personalized preventive interventions

Regarding the current state of personalized prevention, the stakeholders were even more skeptical: on the one hand, predictive tests for some conditions, such as Alzheimer’s disease, already exist, tests for other conditions are in development. On the other hand, there are currently hardly any corresponding interventions that could effectively prevent disease outbreak:

(5) (PI1) An interesting approach is Alzheimer’s, which when combining all of the available parameters allows one to determine to 95 percent who will suffer from Alzheimer’s at a later point. However, to this day, we do not have the possibility of fighting it in any way. So, we do know a lot about this disease […], but even with all of our molecular understanding, we have no chance of interfering properly or halting this process in any way.

### Future perspectives of PM

#### Personalized therapeutic interventions

The assessment of future perspectives of PM showed a similar picture as in the case of assessing the current state of research and clinical use: one stakeholder critically noted that the current focus on PM merely results from an exaggeration and it, thus, will not experience any significant future developments:

(6) (RI) I believe that the whole discussion concerning personalized medicine is experiencing a phase of disillusionment, which is part of the typical cycle that such hypes undergo. I also indeed believe that if it is possible to successfully establish an objective, rational point of view of the actual possibilities, then this disillusionment will increase.

According to this respondent, the lack of good prospects results from the scientifically inadequate reductionist approach of PM. Another stakeholder predicted that the extremely high costs of PM will ultimately render a breakthrough impossible. Most respondents, however, held the opinion that treatment approaches based on *stratification*, i.e. identifying patient subgroups based on biomarkers, in the medium and long term would experience a breakthrough:

(7) (RA) This medicine, I would say, has a future, a very significant future, however, not within the next two years. In fact, if I take the scientists that are working on this seriously, then this is a process that, according to them, will take about another ten years until one can truly say that this is a secure application of medical findings that are evidence-based and confirmed through research.

(8) (PI2) [W]e will move forward step by step. I believe that we will have more and more therapeutic stratification based on the molecular structure of the body.

According to some respondents, the potential of PM consists primarily in improving the choice of drugs as well as their dosage based on diagnostic tests. Moreover, personalized therapeutic measures may provide higher efficacy and efficiency, which, in turn, could result in additional benefits for the patients as well as a positive cost impact on the health care system. However, in order to render such developments possible, more basic, translational, and applied research would be necessary. Due to the vast amount of data gained in the course of such research, good information and data management would be of particular importance:

(9) (BR) The least we can say is that this process will be a long and enduring one. Nevertheless, I still consider it to be possible and achievable. It has a lot to do with the fact, I think, that we can accumulate and merge knowledge better and thus, bring about new findings […]. I think […] momentarily, we are limited by the fact that each individual scientist who works in their respective field does not possess all of the information needed to create an overall concept.

#### Personalized preventive interventions

Compared to personalized treatment, the future perspectives of personalized *prevention* were assessed much more skeptical in almost every interview: several respondents doubted that an adequate medical and scientific foundation of personalized prevention is possible at all. The underlying idea of personalized prevention, however, was predominantly conceived as a vision worth seeking:

(10) (PI1) However, it would be ideal if we could finally move in the direction of prevention by using biomarkers through certain diagnoses. Not just offering treatment for a disease, but rather avoiding the disease. This would be a vision that one should pursue.

### Ethical implications of PM

#### Personalized therapeutic interventions

With respect to personalized *therapeutic measures*, the prevailing ethical concerns were issues of distributive justice due to escalating costs of care which could result in unequal access to PM interventions:

(11) (BR) I just heard that if a breast cancer patient is between the age of 35 and 45 then she will, more or less, have no problems receiving a prescription for [Herceptin]. If she is between the age of 55 and 65, then she will have a major problem getting the prescription and, if she is insured with the AOK [major public health insurance provider in Germany], she will most likely not get the prescription at all. So, it is thus calculated what the life of a 65-year old is worth. Is it worth it to possibly have to spend 50,000 Euros annually for the treatment of this patient? […] What does personalized medicine mean for the equal treatment of patients?

In contrast, some stakeholders expressed the opinion that personalized therapies based on stratification may contribute to the solution of existing distributional issues by reducing inefficiencies. Finally, several respondents expressed the fear that stratification may result in additional risks for patients: as clinical studies on personalized therapeutic measures can be carried out with small patient groups only, drugs might be authorized without being sufficiently tested, especially with respect to possible side effects.

#### Personalized preventive interventions

With reference to personalized *prevention*, most of the respondents mentioned problems of confidentiality, the handling of sensitive genetic data, as well as the possibility of ethnic discrimination or inequalities regarding health insurance and chances on the job market. Furthermore, many stakeholders expressed their worries about a growing attribution of individual responsibility for one’s own health as a consequence of improved means of prevention. Of central concern, however, were potential consequences of prognostic test results on individual well-being:

(12) (HE) A label is established and this label does something to human beings. Basically, the passing on of information is a type of intervention. In the worst case, this leads to fear and low-spirits, depression, and so on.

According to some respondents, this problem is apparent especially in view of possible false-positive prognostic test results.

#### Research on PM

With regard to research, primarily aspects of resource allocation were discussed, in particular allocation of resources into the development of personalized therapeutic approaches. One respondent urged to examine the expected additional benefit in relation to the incurred costs in order to prevent an excessive and unjustified allocation of resources into the research of PM:

(13) (BR) One question that continuously consumes us and that, I personally believe, has not been answered satisfactorily, and where parameters have to be set up, is: is it recommendable, from a health economics perspective, to go this way? If we go ahead with stratification and then […] we end up having to wait for the final answer: did he survive or not? Instead, I would prefer to have parameters beforehand so that I can intervene.

Regarding research practice, the problems of informed consent, privacy of personal data, as well as the right to know/not to know were the focus. Additionally, many interviews centered on the debate on appropriate study designs in the context of approving new drugs, with a common critique of the prevailing use of surrogate parameters:

(14) (RI) We go so far as to say that progression-free survival is not enough, instead, one needs to be able to have a longer life in good quality.

(15) (RA) Especially with regard to cancer therapies, there is hardly any research that is conducted along the lines of these [patient-relevant] outcomes, but instead they are conducted according to surrogate parameters. […] However, this has nothing to do with the patients’ quality of life.

On the other hand, one respondent expressed the opinion that standards for clinical trials are too demanding. As a consequence, clinical trials would require too much time and effort before therapeutic measures can be launched which, in turn, would lead to an unjustified negative impact on patients that could have benefitted from an earlier introduction. Therefore, standards for clinical trials should be scaled down, even if this results in higher risks for patients.

## Discussion

To summarize, all statements concerning the state of PM can be assigned to either of two categories: (I) “development of PM is in its fledgling stages” (optimistic) or (II) “the approach of PM is unsound” (pessimistic), where there is a clear predominance on part of category (I) (15 of 17 respondents). However, even the optimistic respondents assessed the research state of therapeutic measures much more positively than the state of measures of primary prevention.

A similar picture was drawn regarding future perspectives of PM: On the one hand, some stakeholders were generally pessimistic regarding future developments. On the other hand, however, optimistic assessments, which ascribe great potential to personalized *treatment* approaches based on stratification, clearly dominate. Nevertheless, most of the optimistic stakeholders assessed future developments of personalized *prevention* rather pessimistically. A third group of stakeholders felt unable to predict future developments: according to them, the plausibility of PM’s basic idea as well as its additional benefits for patients needs to be proven first in order to render informed predictions possible. In their opinion, PM’s cost impact on the health care system should also be clarified before making forecasts regarding its development.

It has to be noticed, however, that at least some of the assessments of PM’s current and future perspectives seem to follow the respective stakeholder’s interests: one representative of the reimbursement institutions, for instance, assesses PM pessimistically as “a propagandistic idea by the pharmaceutical industry” (interview passage (2)). A representative of patient organizations, on the other hand, grounds his pessimistic evaluation on the suspicion that “the pharmaceutical industry […] is slowing the whole process [of developing PM] down” due to economic considerations (4). Representatives of the pharmaceutical industry, in turn, assess PM, especially its future perspectives, much more optimistic (8), even with regard to personalized prevention (10). These exemplary statements seem to represent the actual competitive situation in the German healthcare system: whereas pharmaceutical companies in recent years increasingly changed their strategy towards development and marketing of PM [14], patient organizations as well as reimbursement institutions are taking a critical look at exactly these changes.

In contrast to such competitively motivated assessments, it is striking that almost all respondents agreed in exclusively alluding to the field of oncology as regarding future perspectives of therapeutic PM measures. Only one expert additionally referred to personalized approaches of pain management, another explicitly demanded research in medical areas outside oncology. This is remarkable as approaches of PM increasingly are discussed outside oncology [3]. Besides, in Germany several interventions outside oncology are already established, e.g. agents for treating epilepsy, HIV/AIDS or multiple sclerosis [11]. In contrast to such developments, the respondents’ focus suggests that the most significant progress of PM can probably be expected in oncology – and that the development of PM in other medical field might be neglected. A possible reason for this is the fact that PM is a rather heterogeneous field of development and application. As a consequence, the respondents’ focus on oncology is probably due to the specific composition of our sample, i.e. the respondents’ specific working fields and personal interests.

When turning to the ethical implications of PM, the participants addressed concerns in PM’s various fields both on the individual and on the societal level as shown in Table 2.

**Table 2:** **Stakeholders**’ **ethical concerns by fields of PM**

		**Fields of PM**		
		***Research***	***Clinical Use *( *Health Care *)**	
			**Prevention**	**Treatment**
**Ethical Concerns**	*Individual Level*	Informed Consent for Biomarker Studies	Impact on Individual Well-Being	Higher Risks for Patients
		Confidentiality & Data Protection	Confidentiality & Data Protection	
		Right to Know/Right not to Know	Right to Know/Right not to Know	
		Study Designs (Patient Relevant Outcomes)	Increased Individual Responsibility for Health	
	*Societal Level*	Allocation of Resources	(Genetic) Discrimination	Distribution

It is of interest that no concerns were mentioned that exclusively arise in the field of PM, but rather issues that are well-known from other contexts, such as biobanking, (conventional) genetic diagnostics, and other fields of biomedical research. Besides, the respondents did neither mention (potential) distributional issues regarding personalized preventive measures nor did they allude to concerns of data protection, right to know/not to know or potential (genetic) discrimination regarding personalized therapeutic measures. Concerning the issues of data protection as well as the right to know/not to know, it is intriguing that another German interview study found that experts in the field of PM pointed exactly to these issues [10]. It is, however, important to realize that the study’s sample (n=19) consisted exclusively of researchers and clinicians involved in the same research group working on PM in the field of colorectal cancer. In contrast, the ratio of researchers (including *basic* researchers) and clinicians was 5 out of 17 in our sample. This difference in sample composition might explain the different focus of the involved experts regarding ethical concerns in the field of PM. It has to be noticed, however, that the results of the two studies generally are difficult to compare as we used a different methodological approach with open questions and no concrete PM-example as stimulus (the experts in [10] were asked explicitly about the implications of their own development, the Rectumchip).

Apart from these methodological considerations, it is particularly the neglect of potential discrimination resulting from personalized therapeutic measures that is striking: after all, disadvantages for certain patient subgroups are to be feared as a consequence of secondary information regarding the prognosis and efficacy of treatments gained by diagnostic tests. This becomes clear by analyzing the praxis of applying the agent Trastuzumab (Herceptin®) for treatment of breast cancer. The drug, especially when applied in combination with chemotherapy or as an adjuvant (postoperative) treatment, significantly improves the response rate and the progression-free as well as overall survival rate in patients with an overexpression of the HER2/neu receptor (HER2/neu positive) as compared to HER2/neu negative patients [15-19]. For this reason, in Germany in the year 2000, treatment with Trastuzumab has been tied down to an HER2/neu positive test result [11]. This practice, however, means to categorize breast cancer patients as either “good responders”, “non-responders” or “difficult to treat“, which is at least a *potential* cause of discrimination: such categorizations eventually result in restricting access for the subpopulations labeled as “non-responder” or “difficult to treat” to health care interventions and health insurances or disadvantaging them in other areas, for instance on the job market [20-23]. As was the case for the respondents’ focus on oncology, a possible reason for their neglect of these issues probably lies in the specific composition of our sample: respondents may had only such agents in mind that are positively connoted (e.g. Imatinib) [24], while not bearing in mind negatively connoted ones (e.g. Cetuximab) [25].

In summary, the results of our study show at least two biases in the discourse about PM: first, the respondents’ almost exclusively focus on the field of oncology and second, their neglect of certain ethical concerns. Needless to say, these biases indicate a fundamental problem of the ethical discourse on PM: ethical concerns in many cases apparently do not adequately reflect PM’s current state of research and clinical usage as well as the prognosis of its future developments. This impression also is supported by the fact that, although most respondents assessed future developments of personalized prevention rather pessimistically, they nevertheless located a multitude of potential ethical issues in this context. On the other hand, relatively few concerns were mentioned regarding personalized therapeutic approaches even though their future developments were assessed much more optimistically. As regards future developments of PM the ethical evaluation seems to follow the rule: the less likely its application (i.e. a pessimistic assessment), the more problematic a PM measure is assessed. The more likely its application (i.e. an optimistic assessment), on the other hand, the less problematic it is evaluated. As a consequence, ethical discourse on PM runs the risk of debating (currently) less relevant ethical concerns while neglecting more relevant ones.

## Conclusions

Against this background, our findings strongly suggest a re-adjustment of the ethical debate on PM. A *constructive* ethical monitoring must ensure to include *only*, nevertheless *all* of the actual and/or potential concerns that are *ethically relevant* in order to allow balancing them against the actual and potential ethically relevant benefits of PM measures. This is the only way to ensure an ethical monitoring of PM’s developments and clinical usage, which carves out ethical issues while not, on the other hand, retarding reasonable developments and applications.

Yet, as has been noted above, PM is a rather heterogeneous field of development and application. Therefore, ethically relevant concerns differ decisively with regard to different PM measures. On this account, an overall assessment of PM’s (potential) ethical issues is impossible. Rather, an adequate evaluation strategy requires assessing each personalized approach separately with regard to its actual or potential concerns. Furthermore, it is necessary to re-focus towards more likely fields of application. Consequently, in order to determine ethically relevant concerns of a certain PM intervention, its respective current state of research and clinical usage as well as its expected future developments has to be analyzed and evaluated. It generally holds: the less likely that a PM measure be applied, the less relevant are the concerns associated with it. The more likely that a PM measure be applied, the more relevant are the concerns associated with it. However, the (potential) impact of an ethical concern on individual patients as well as the healthcare system and the society is also important for evaluating its relevance. With respect to this aspect it holds: the less critical a concern associated with a PM measure, the less relevant it is for evaluating the measure. The more critical a concern, the more relevant it is. Needless to say, evaluating a (potential) impact as highly critical may makes is necessary to regard concerns as relevant that are associated with PM measures whose application is unlikely. After all, the quality of an ethical concern may overrule the results of evaluating the current state of research and clinical usage as well as the expected future developments of an PM measure. Thus, the more critical an ethical concern associated with an PM measure is, the less important is the likelihood of the measures’ application for evaluating the concerns’ relevance and vice versa. It is obvious, however, that this criterion again increases the risk of debating irrelevant ethical concerns, either if concerns are evaluated as highly critical which are very unlikely to occur, or if critical concerns are associated with PM measures whose application is very unlikely. In order to avoid such debates, both (potential) ethical concerns and the application of a PM measure have to be plausible to a certain degree in order to qualify a concern as relevant [26]. If this is guaranteed, we claim that ethical concerns should have priority in the ethical discourse according to our criteria of relevance. As none of the potential concerns seems to arise exclusively in the context of PM, they can be approached by referring to standard bioethics debates and applying accordant approaches.

### Limitations

Like in all qualitative research, the empirical results of this study are not representative, but rather dependent on the particular sample underlying the analysis. Consequently, in that the need for a re-accentuation of the ethical discourse on PM is inferred from the empirical results of a qualitative study, it is to be understood as a hypothesis, which has to be supported by further quantitative studies, e.g. on the ethical concerns mentioned by a representative sample of stakeholders in the German healthcare system.

## Competing interests

The authors declare that they have no competing interests.

## Authors’ contributions

SS and GM designed the study, SS acquired the data. SS and GM carried out data analysis as well as interpretation. SS drafted the manuscript, GM revised it. Both authors read and approved the final manuscript.

## Pre-publication history

The pre-publication history for this paper can be accessed here:

http://www.biomedcentral.com/1472-6939/14/20/prepub

## Supplementary Material

Additional file 1 Coding Tree. Click here for file
