# Supplementary material for: Re-focusing the ethical discourse on personalized medicine: a qualitative interview study with stakeholders in the German healthcare system
Source: BMC Med Ethics. 2013 May 24;14:20. doi: 10.1186/1472-6939-14-20 (PMC3681604; doi:10.1186/1472-6939-14-20)
Supplement: Additional file 1 — Coding Tree. [file 1472-6939-14-20-S1.doc]

**Additional file: Coding Tree**

Assessment of the current state of IHC

IHC is at the stage of development

Approach of IHC is inadequately reductionist

(So far) lack of medical/scientific foundation

Development could be further advanced

Pharmaceutical industry slows down development deliberately

Pharmaceutical industry has no interest in a fast development of IHC

Problem: management of information

Individualized prevention is a vision

(So far) lack of medical/scientific foundation

(So far) lack of measures of intervention

Diagnostic testing/treatment is at the stage of development

(So far) lack of medical/scientific foundation

Complexity of biological organisms

Several stratified measures are in use

(So far) inadequate additional benefit

Causes for current presence

Scientific interests

Economic interests of pharmaceutical industry

Pressure to innovate on the pharmaceutical industry

Prognosis of future development of IHC

Prognosis is impossible

Proof of the approach’s plausibility is necessary

Proof of additional benefit for patients is necessary

Medical/scientific foundation is necessary

Cost Impact of IHC on the health care system is unsettled

IHC has no (significant) future

Sufficient medical/scientific foundation is impossible

Approach is reductionist

Literal individualization of treatment is impossible

Development of attention in accordance to hype cycle

Individualized prevention has no/little future

Sufficient medical/scientific foundation is impossible

Compliance is questionable

Individualized treatment has no/little future

Negative cost impact on the health care system

Development of individualized treatment will take place in small increments

Dependence on management of information

Wide usage will be possible medium to long term

Stratification will advance further

Diagnostics will obtain higher priority

Individualized treatment has great potential

Positive cost impact on the health care system

Improvement of conditions for treatment decisions

Additional benefit for patients

Better efficacy

Better efficiency

Ethical concerns regarding IHC

Research

Negative effects of study requirements on patients

Study designs (surrogate parameters vs. patient relevant outcomes)

Informed consent

Right to Know/Right not to Know

Data protection

Allocation of resources into research

Diagnostic tests/treatment

Distribution of therapeutic measures

Negative cost impact on the health care system

Stratification bears additional risks on patients

Prognostic tests/prevention

Genetic discrimination

Ethnic discrimination

Discrimination on the job market

Discrimination on the insurance market

Right to Know/Right not to Know

Data protection

Impact on individual well-being

False positive tests

Lack of measures of intervention

Augmented attribution of responsibility/duty for one's own health
